# Fluid Simulations Accelerated With 16 Bits: Approaching 4x Speedup on A64FX by Squeezing ShallowWaters.jl Into Float16

**DOI:** 10.1029/2021MS002684

**Published:** 2022-02-11

**Authors:** Milan Klöwer, Sam Hatfield, Matteo Croci, Peter D. Düben, Tim N. Palmer

**Affiliations:** ^1^ Atmospheric, Oceanic and Planetary Physics University of Oxford Oxford UK; ^2^ European Centre for Medium‐Range Weather Forecasts Reading UK; ^3^ Mathematical Institute University of Oxford Oxford UK

**Keywords:** low precision, floating‐point numbers, climate models, rounding errors, hardware acceleration

## Abstract

Most Earth‐system simulations run on conventional central processing units in 64‐bit double precision floating‐point numbers Float64, although the need for high‐precision calculations in the presence of large uncertainties has been questioned. Fugaku, currently the world's fastest supercomputer, is based on A64FX microprocessors, which also support the 16‐bit low‐precision format Float16. We investigate the Float16 performance on A64FX with ShallowWaters.jl, the first fluid circulation model that runs entirely with 16‐bit arithmetic. The model implements techniques that address precision and dynamic range issues in 16 bits. The precision‐critical time integration is augmented to include compensated summation to minimize rounding errors. Such a compensated time integration is as precise but faster than mixed precision with 16 and 32‐bit floats. As subnormals are inefficiently supported on A64FX the very limited range available in Float16 is 6 × 10^−5^ to 65,504. We develop the analysis‐number format Sherlogs.jl to log the arithmetic results during the simulation. The equations in ShallowWaters.jl are then systematically rescaled to fit into Float16, using 97% of the available representable numbers. Consequently, we benchmark speedups of up to 3.8x on A64FX with Float16. Adding a compensated time integration, speedups reach up to 3.6x. Although ShallowWaters.jl is simplified compared to large Earth‐system models, it shares essential algorithms and therefore shows that 16‐bit calculations are indeed a competitive way to accelerate Earth‐system simulations on available hardware.

## Key Points

- The first fluid circulation model entirely based on 16‐bit instead of conventional 64‐bit calculations approaches 4x speedups on hardware
- Systematic rescaling squeezes all calculations into the very limited range of Float16, making use of 97% of the available numbers
- Compensated summation in the precision‐critical time integration minimizes rounding errors from Float16 and is faster than mixed precision

## 1 Introduction

Recently several numerical weather prediction models have moved away from 64‐bit double‐precision floating‐point numbers (Float64) for higher computational efficiency in lower precision (Govett et al., 2017; Maynard & Walters, 2019; Nakano et al., 2018; Rüdisühli et al., 2013; Váňa et al., 2017). Both 32‐bit single‐precision floating‐point numbers (Float32) and Float64 formats are widely available for high‐performance computing. However, support for 16‐bit arithmetic is only available on mainstream hardware for a few years, due to the demand for low precision by deep learning. The transition for an existing application toward 16 bits is challenging: Rounding errors from low precision have to be controlled and a limited range of representable numbers cannot be exceeded without causing often catastrophic under and overflows. But the potential performance gains are promising with 4x speedups compared to 64‐bit calculations, not to mention the reduced energy consumption.

The current boom in machine learning applications is supported by advances in microprocessors. Instead of conventional central processing units (CPU), graphic and tensor processing units (GPU, TPU) are used (N. Jouppi et al., 2018; N. P. Jouppi et al., 2017, 2018; Steinkraus et al., 2005), which are better suited for the workloads of machine learning. While most supercomputers are based on Intel CPUs with the x86‐64 architecture (Dongarra & Luszczek, 2011, p. 500), many new installations have GPUs or alternative microprocessor architectures (Zheng, 2020). The trend is toward heterogeneous computing with specialized hardware, which is both a challenge and an opportunity for weather and climate models (Bauer, Dueben, et al., 2021; Bauer, Stevens, & Hazeleger, 2021; Lawrence et al., 2018). Fugaku, the world's fastest supercomputer as of 2020, is based on Fujitsu's A64FX processors with ARM architecture (Odajima et al., 2020; Sato et al., 2020). The A64FX also implements 16‐bit half‐precision floating‐point numbers (Float16, 1 sign, 5 exponent and 10 mantissa bits) and Fujitsu promises a four‐fold increase in the number of floating‐point operations per second (FUJITSU, 2020; Odajima et al., 2020).

The IEEE‐754 standard on floating‐point arithmetic (IEEE, 1985, 2008) defines floating‐point numbers with 16, 32, and 64 bits. Programming languages and computational hardware implement these formats and various terms such as binary16/32/64 or half/single/double are frequently used. Here we use the terms Float16, Float32, and Float64 from the Julia programming language (Bezanson et al., 2017) for both definition and implementation interchangeably.

Alternatives such as BFloat16 (Burgess et al., 2019; Kalamkar et al., 2019), minifloats (Fox et al., 2020), logarithmic fixed‐point numbers (Johnson, 2018, 2020; Sun et al., 2020), posits (Chaurasiya et al., 2018; Gustafson & Yonemoto, 2017; Klöwer et al., 2019, 2020; Langroudi et al., 2019; Zhang & Ko, 2020) and stochastic rounding (Croci & Giles, 2020; Hopkins et al., 2020; Mikaitis, 2020; Paxton et al., 2021) have been investigated, but most of these are not available on standard supercomputing hardware. Currently only floats (and integers) enjoy a widely available support in terms of hardware, libraries and compilers that ultimately make it possible to execute complex computational applications.

The use of low‐precision number formats is motivated as in the presence of large uncertainties in the climate system rounding errors are masked by other sources of error (Palmer, 2015). Typical rounding errors from high‐precision calculations are many orders of magnitude smaller than errors in the observations, from coarse resolution or underrepresented physical processes. Low‐precision calculations are therefore, at least in theory, sufficient without a loss in accuracy for a weather forecast or a climate prediction. Emulated in parts of weather and climate models, Float16 has been shown to be a potential route to accelerated simulations (Chantry et al., 2019; Dawson et al., 2018; Hatfield et al., 2019; Klöwer et al., 2020).

Although weather and climate model data often comes with large uncertainties, many intermediate calculations inside a model simulation require a higher precision. Time integration is often a precision‐critical part of numerical simulations of dynamical systems (Dawson et al., 2018; Klöwer et al., 2020). Stability constraints require small time steps such that tendencies are often several times smaller than the prognostic variables (Courant et al., 1967). Adding the two yields a loss of precision from the tendency as small increments can only be poorly resolved in low precision (S. Gill, 1951; Kahan, 1965; Møller, 1965). In extreme cases this can lead to a model stagnation (Croci & Giles, 2020), and is often dealt with using mixed‐precision approaches (Dawson et al., 2018; Klöwer et al., 2020; Tintó Prims et al., 2019), where the tendencies are computed in low‐precision, but converted to a high‐precision format before addition. This is beneficial as a large share of computing time is accelerated with low precision, while precision‐critical operations are kept in high precision.

Precision loss in calculations can be analyzed with a variety of available tools, like FPBench (Damouche et al., 2017), CADNA (Jézéquel & Chesneaux, 2008), Verrou (Fevotte & Lathuilière, 2019), and Verificarlo (Denis et al., 2016). Such tools are often either based on interval arithmetic, providing rigid rounding error bounds, or on stochastic arithmetic to assess the rounding error growth. While these can be useful to identify the minimal decimal precision for simulating chaotic systems, analyzing the limited dynamic range of low‐precision number formats is largely unaddressed in these tools.

Here, we present, to our knowledge, the first fluid circulation model that runs entirely in hardware‐accelerated 16‐bit floats on the ARM architecture‐based microprocessor A64FX. Strategies are presented to solve precision and range issues with 16‐bit arithmetic: In Section 2 we scale the shallow water equations, and an appropriate scale is found with the newly developed analysis‐number format Sherlogs.jl, which is introduced in Section 3. A compensated time integration is presented in Section 4 to minimize precision issues. Section 5 analyses the rounding errors of Float16 in ShallowWaters.jl and benchmarks the performance compared to Float64. Section 6 discusses the results.

## 2 Scaling the Shallow Water Equations

The shallow water equations describe atmospheric or oceanic flow idealized to two horizontal dimensions. They result from a vertical integration of the Navier‐Stokes equations (A. Gill, 1982; Vallis, 2006) and are simplified but representative of many weather and climate models, which are usually solved with many vertically coupled horizontal layers. They describe the time evolution of the prognostic variables velocity **u** = (*u*, *v*), and interface height *η* in the following form

(1) $$\begin{array}{lll} \partial_{t}u & + & u\cdot \nabla u+fz\times u=-g\nabla \eta +\nu_{B}\nabla^{4}u-ru+F\\ \partial_{t}\eta & + & \nabla \cdot \left(uh\right)=0\\ \partial_{t}q & + & u\cdot \nabla q=-\tau \left(q-q_{0}\right) \end{array}$$

defined over a rectangular domain with zonal and meridional coordinates *x*, *y* of size *L*_*x*_ = 8,000 km, *L*_*y*_ = 4,000 km, respectively. The domain is a zonal channel with boundary conditions being periodic in *x*. The channel setup is motivated by zonal flows like the Antarctic Circumpolar Current but highly idealized (Jansen, Adcroft, et al., 2015; Jansen, Held, et al., 2015).

The non‐linear momentum advection is u⋅∇u. The Coriolis force is $f\hat{z}\times u=\left(-fv,fu\right)$ with the Coriolis parameter *f* using a *β*‐plane approximation at 45°N. The pressure gradient −*g*∇*η* scales with a reduced gravitational acceleration *g* = 0.01 ms^−2^ to represent baroclinic ocean/atmosphere dynamics (A. Gill, 1982). The zonal wind forcing **F** = (*F*_*x*_, 0) is a meridional shear $F_{x}=F_{0}\;\sin\;(\omega t)\;\tanh\;(2\pi (L_{y}^{-1}-\frac{1}{2}))$ which reverses seasonally (*ω* ^−1^ = 365 days). Lateral diffusion of momentum is described by *ν*_*B*_∇^4^ **u**, with biharmonic viscosity coefficient *ν*_*B*_. Linear bottom friction is represented by −*r* **u** which decelerates the flow at a time scale of *r* ^−1^ = 300 days. The equation for interface height *η* is the shallow water‐variant of the continuity equation, ensuring conservation of volume (and mass as the density *ρ* is constant). The layer thickness is *h* = *η* + *H* of a fluid with depth *H* at rest. Several meridional ridges on the seafloor trigger instabilities in the zonal flow, but they are small compared to the fluid depth. The shallow water equations are complemented with an advection for the passive tracer *q*, which is stirred by the flow through u⋅∇q and slowly (*τ* ^−1^ = 100 days) relaxed back to a reference *q*_0_.

In order to control the range of numbers occurring in the simulation, the shallow water equations are scaled with a multiplicative constant. The evaluation of linear terms is not affected, but the non‐linear terms involve an unscaling. The same constant *s* is chosen for zonal velocity *u* and meridional velocity *v*, such that $\hat{u}=su$ and $\hat{v}=sv$. Additionally, we use dimensionless spatial gradients $\hat{\partial_{x}}=\Delta x\partial_{x},\hat{\nabla }=\Delta x\nabla$, etc. by scaling the equations with the grid spacing Δ*x*. For simplicity, we use the same Δ*x* in *x* and *y*‐direction but generalization to less regular grids is possible. The grid spacing Δ*x* is then combined with the time step $\hat{\Delta t}=\frac{\Delta t}{\Delta x}$ and $\hat{\partial_{t}}=\Delta x\partial_{t}$. Due to the fourth‐order gradient in the viscosity, we scale its coefficient as $\hat{\nu_{B}}=\Delta x^{-3}\nu_{B}$. Using the potential vorticity *h* ^−1^(*f* + *ζ*), with the relative vorticity *ζ* = *∂*_*x*_ *v* − *∂*_*y*_ *u*, and the Bernoulli potential $\frac{1}{2}\left(u^{2}+v^{2}\right)+g\eta$, the shallow water equations can be written into a scaled form as

(2) $$\begin{array}{lll} \hat{\partial_{t}}\hat{u} & = & \frac{[s\Delta xf]+\hat{\zeta }}{\hat{h}}\frac{\hat{v}\hat{h}}{s}-\hat{\partial_{x}}\left(\left[\frac{1}{2s}\right]\left({\hat{u}}^{2}+{\hat{v}}^{2}\right)+\left[\frac{sg}{s_{\eta }}\right]\hat{\eta }\right)+\hat{\nu_{B}}{\hat{\nabla }}^{4}\hat{u}-\left[r\Delta x\right]\hat{u}+\left[s\Delta xF_{x}\right]\\ \hat{\partial_{t}}\hat{v} & = & -\frac{[s\Delta xf]+\hat{\zeta }}{\hat{h}}\frac{\hat{u}\hat{h}}{s}-\hat{\partial_{y}}\left(\left[\frac{1}{2s}\right]\left({\hat{u}}^{2}+{\hat{v}}^{2}\right)+\left[\frac{sg}{s_{\eta }}\right]\hat{\eta }\right)+\hat{\nu_{B}}{\hat{\nabla }}^{4}\hat{v}-\left[r\Delta x\right]\hat{v}+\left[s\Delta xF_{y}\right] \end{array}$$

Square brackets denote pre‐computed constants and only the volume fluxes *uh*, *vh* have to be unscaled on every time step. As the volume fluxes are quadratic terms, the evaluation of $\hat{u}\hat{h}$ scales as *s* ^2^, which therefore has to be partly unscaled with *s* ^−1^. The continuity equation is rescaled with *s*_*η*_, that is, $\hat{\eta }=s_{\eta }\eta$ as well as $\hat{h}=\hat{\eta }+s_{\eta }H$, and the tracer advection equation is rescaled with *s*_*q*_, so that $\hat{q}=s_{q}q$

(3) $$\begin{array}{lll} \hat{\partial_{t}}\hat{\eta } & = & -\hat{\partial_{x}}\left(\frac{\hat{u}\hat{h}}{s}\right)-\hat{\partial_{y}}\left(\frac{\hat{v}\hat{h}}{s}\right)\\ \left[s\hat{\partial_{t}}\right]\hat{q} & = & \left(-\hat{u}\hat{\partial_{x}}\hat{q}-\hat{v}\hat{\partial_{y}}\hat{q}\right)-\left[\tau \Delta x\right]\left(\hat{q}-\hat{q_{0}}\right) \end{array}$$

ShallowWaters.jl solves these scaled shallow water equations with second order finite differencing on a regular, but staggered Arakawa C‐grid (Arakawa & Lamb, 1977). The advection of potential vorticity uses the energy and enstrophy‐conserving scheme of Arakawa and Hsu (1990). The tracer advection equation for *q* is solved with a semi‐Lagrangian advection scheme (Diamantakis & Váňa, 2021; Smolarkiewicz & Pudykiewicz, 1992). This scheme calculates a departure point for every arrival grid point one time step ago. The tracer field is then interpolated onto the departure point, which is used as the tracer concentration at the arrival point for the next time step. More details on the implementation of the semi‐Lagrangian advection scheme is described in Klöwer et al. (2020). The time integration of ShallowWaters.jl is discussed in Section 4.

## 3 Choosing a Scale With Sherlogs

**Figure 1:**
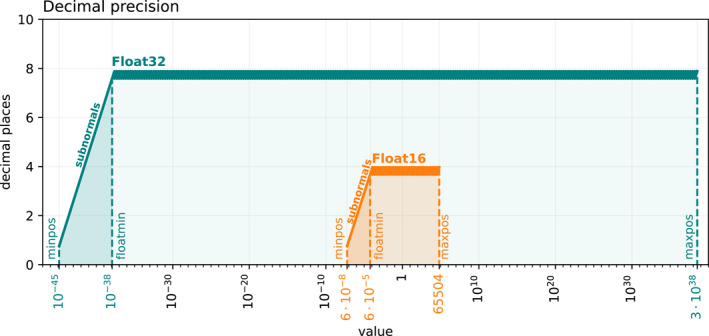
Decimal precision of Float16 and Float32 over the range of representable numbers. The decimal precision is worst‐case, that is, given in terms of decimal places that are at least correct after rounding (see Appendix A). The smallest representable number *minpos*, the smallest normal number *floatmin* and the largest representable number *maxpos* are denoted with vertical dashed lines. The subnormal range is between minpos and floatmin.

The scaling of equations has to be implemented carefully when using number formats with a limited dynamic range, such as Float16 (Figure 1). Subnormals for Float16 are in the range of approximately 6 × 10^−5^ and 65,504 (see Appendix A). Subnormals are inefficiently supported on some hardware, such that their occurrence causes large performance penalties. This reduces the available range of Float16 even further and a simulation has to fit as best as possible in the remaining nine orders of magnitude between 6 × 10^−5^ and 65,504. A single overflow, that is, a result above 65,504, yields infinity, effectively aborting the simulation. Understanding the range of numbers that occur in all operations and ideally in which lines of the code is therefore very important. For most algorithms this is very difficult to achieve unless the numbers are directly measured within the simulation.

We therefore developed the analysis‐number format Sherlogs. Sherlog16, for example, uses Float16 to compute, but after every arithmetic operation the result is also logged into a bit pattern histogram. Running a simulation with Sherlogs will take considerably longer due to the overhead from logging the arithmetic results, which can be obtained in the form of a bit pattern histogram upon completion. The bit pattern histogram will reveal information such as the smallest and largest occurring numbers or how well an algorithm fits into a smaller dynamic range. An example usage of Sherlogs in Julia is given

1 julia> using ShallowWaters, Sherlogs       # load packages
2 julia> # run ShallowWaters with Sherlog16 which logs all arithmetic results
3 julia> run_model(Sherlog16)                # use Sherlog16 as number format
4
 
5 julia> get_logbook()                       # retrieve the bitpattern histogram
6 65536‐ element LogBook(1112720887, 1484631, 1378491, 1024411, ... , 0, 0, 0)

Using Sherlog16 as the first argument of run_model runs ShallowWaters.jl with Float16 but also logs the bit pattern of every arithmetic result into a logbook of length 2^16^ = 65,536 to create a bit pattern histogram. Sherlogs are implemented in the package Sherlogs.jl, which makes use of the type‐flexible programming paradigm in Julia (Bezanson et al., 2017). A function is written in an abstract form, which is then dynamically dispatched to the number format provided and compiled just‐in‐time. Such a number format can therefore be, for example, Float64 or Float16, but also any user‐defined number format such as Sherlogs.

**Figure 2:**
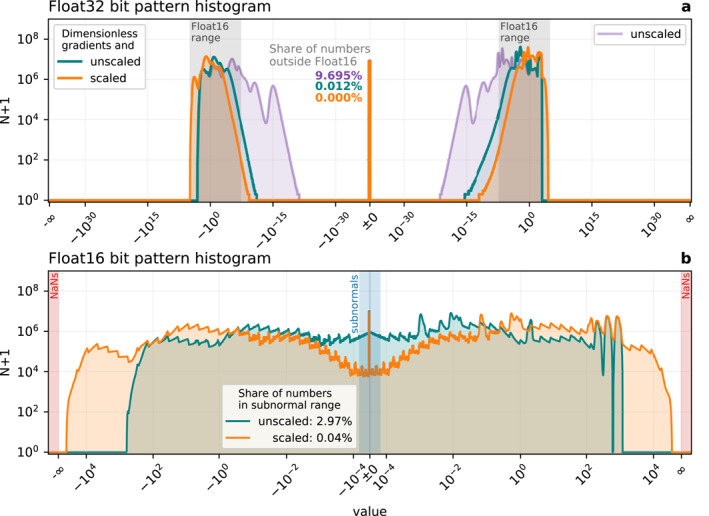
Bitpattern histogram of all arithmetic results in ShallowWaters.jl. (a) 200‐day simulation at Δ*x* = 20 km based on Float32 arithmetic. The share of numbers outside the Float16 range (gray shading) are color‐coded to the respective histograms. (b) as (a) but based on Float16. Bitpattern histograms are created with Sherlogs.jl. The logarithmic *y*‐axis denotes the number of occurrences *N* of the respective bitpattern during the simulation. The histograms span all available bitpatterns (0x0000 to 0xffff in hexadecimal) in the respective formats evenly but are sorted and relabeled with the corresponding values for readability. The range of bitpatterns that are subnormals or interpreted as Not‐A‐Number (NaN) are marked. Bit pattern histograms are without compensated time integration.

An appropriate scaling has to be chosen for a given set of parameters. The bit pattern histogram of the entirely unscaled shallow water equations simulated with Float32 reveals range issues that would arise with Float16 (Figure 2a). A large share of 10% of the arithmetic results would be below the representable range of Float16. Consequently, running the model without any scaling modifications in Float16 would round many numbers to 0, causing so‐called underflows that deteriorate the simulated dynamics (Klöwer et al., 2020). Most of these underflows occur in the calculation of gradients, which consequently have to be non‐dimensionalized as previously suggested (Klöwer et al., 2019). This also largely removes a resolution‐dependence of the bit pattern histograms, such that Float16 simulations are possible across a wide range of resolutions. Dimensionless gradients are a major improvement to fit ShallowWaters.jl into the available range with Float16, yet 3% of the arithmetic results are subnormals (Figure 2b). On A64FX a flag can be set to avoid the performance penalty from subnormals by flushing every occurring subnormal to zero. The smallest representable number is then 6.104 × 10^−5^.

In order to systematically identify occurrences subnormals, or any other range, we use the analysis format DrWatson, which is also implemented in the Sherlogs.jl package. An example usage is given

8 julia> # run ShallowWaters with DrWatson16 recording a stack trace when *f* = true
9 julia> *f* (*x*) = 0 < abs(*x*) < floatmin(Float16)       # true for subnormals
10 julia> run_model(DrWatson16{f})                     # use DrWatson16 as number format
11
 
12 julia> get_stacktrace(1)                            # retrieve first stack trace
13 3‐ element Vector{Base.StackTraces.StackFrame}:
14 * at DrWatson16.jl:52 [inlined]                     # subnormal occurred in *
15 caxb!(...) at time_integration.jl:320               # inside this function
16 time_integration(...) at time_integration.jl:82     # called from here

DrWatson16{f} uses Float16 but also records a stack trace (a list of calling functions and respective lines of code) every time the function *f*(*x*) evaluates to true with the arithmetic result *x*. Here, a subnormal arises in a multiplication (* in line 14 here) in line 320 of the code in script time_integration.jl.

Using the DrWatson number format from Sherlogs.jl identifies the addition of the tendencies to the prognostic variables *u*, *v*, *η* as prone to produce subnormals. We therefore increase the scales *s*, *s*_*η*_ to scale up the prognostic variables and consequently their tendencies. Choosing *s* = 2^6^, *s*_*η*_ = 1 reduces the amount of subnormals to 0.04%, while leaving about a factor two head space between the largest occurring numbers (about 30,000) to avoid overflows beyond 65,504 (Figure 2b). The compensated time integration (Section 4) increases this share to about 0.2%.

A scale smaller than *s* = 2^6^ can be chosen in case the risk of an overflow should be further reduced. In our application, the scale can be any number up to about 100, beyond which overflows abort the simulation. A power of two as scaling factor is only chosen to not introduce additional rounding errors in the scaling of the initial conditions, which, however, are negligible compared to the rounding errors in the time integration (Section 5).

The idealized tracer in ShallowWaters.jl takes values in (−1, 1), so we scale this variable by *s*_*q*_ = 2^15^ in order to use most of the Float16 range. This is to allow as many bit patterns as possible for the interpolation in the semi‐Lagrangian advection scheme, which uses non‐dimensional departure points on a locally relative grid for 16‐bit arithmetic, as described in Klöwer et al. (2020).

Consequently, the fully scaled shallow water equations are squeezed well into Float16, making near‐optimal use of the available bit patterns, of which only 3% are unused (NaNs excluded). In contrast, a simulation with Float32 does not make use of at least 81% of available bit patterns (Figure 2), assuming that for a simulation run long enough all bit patterns within the used range occur eventually. Extrapolating this to Float64 with a representable range of 5 × 10^−324^ to 2 × 10^308^ the share of unused bit patterns is at least 97.5%. This computational inefficiency can be overcome with 16‐bit number formats and systematic scaling as presented here. However, scaling leaves the precision issues with low‐precision formats unaddressed, for which we present a technique in the next section.

## 4 A Compensated Time Integration

To minimize the precision loss in the time‐integration, we adopt compensated summation as an alternative approach to mixing precision. Compensated summation is a simple, yet powerful technique that prevents the accumulation of rounding errors in the computation of large sums. Since the addition of multiple terms is ubiquitous in scientific computing, compensated summation can be used to improve the accuracy of many algorithms such as numerical linear algebra operations, integration or optimization. Here we use compensated summation to augment the resilience to rounding errors of our half‐precision time‐stepping method.

The first version of compensated summation was used by S. Gill (1951) in a Runge‐Kutta integrator scheme in fixed‐point arithmetic, and the idea was subsequently extended to floating‐point arithmetic by Kahan (1965), Møller (1965), and others (Higham, 1993; Linnainmaa, 1974; Vitasek, 1969). That we are aware of, our paper is the first work in which compensated summation is used in a fluid circulation model with 16‐bit arithmetic.

To understand compensated summation, consider the following naïve algorithm for the summation of all the entries of a length‐*n* vector *a*_*i*_, *i* = 1, …, *n*

1    sum = 0              # variable to store the sum
2    for a_i_ in a          # loop over all elements of a
3       sum = sum + a_i_    # accumulate each element into sum
4    end
5    return sum

This algorithm is prone to rounding errors, which accumulate at a rate proportional to *n* Higham (1993). Furthermore, the algorithm might cause stagnation, a phenomenon for which the partial sum becomes too large, causing each subsequent addition to be neglected due to rounding. Compensated summation offers a much better alternative at the cost of introducing an additional compensation variable c.

1    c =  0                     # compensation, initially 0
2    sum = 0                    # variable to store the sum
3    for a_i_ in a                # loop over all elements of a
4        a_ic_ = a_i_ − c           # compensate error from previous iteration
5        temp = sum + a_ic_       # add next element of a, but store in temp
6        c = (temp‐ sum) − a_ic_  # rounding error from sum +  a_ic_

7        sum = temp             # copy addition back to sum
8    end
9    return sum

At infinite precision, the compensation c will remain 0. At finite precision, however, calculating c = (temp‐sum) − aic will estimate the rounding error in the addition sum + aic and subsequently attempt to compensate for it in the next iteration through aic = ai − c. For base‐2 floating‐point arithmetic we have exactly sum + ai = temp + c, that is, the compensation variable c correctly captures the rounding errors in the addition. Compensated summation prevents the rounding errors from accumulating, and the overall summation error will stay a mere multiple of machine precision (Higham, 1993). Overall, the compensation c can be interpreted as a storage variable for rounding errors and effectively prevents rounding errors in the summation from growing beyond machine precision accuracy.

Compensated summation is especially useful in settings in which the order of summation cannot be manipulated to prevent rounding error growth. Time integration schemes, for which the state variables are updated sequentially, are especially amenable to augmentation by compensated summation. Over a time period *T* the number of terms to be added scales as *T*Δ*t* ^−1^, proportional to one for each time step. The naïve algorithm would cause rounding errors to grow like $O\left(T\Delta t^{-1}\varepsilon \right)$ with machine epsilon *ɛ*, causing errors to counter‐intuitively grow as the time‐step is refined. With compensated summation the rounding errors will stay O(ε).

ShallowWaters.jl uses the 4‐th order Runge‐Kutta scheme (Butcher, 2008) to integrate the non‐dissipative terms in time: The momentum advection **u · ∇u;** the Coriolis force *f* **z** × **u**; the pressure gradient −*g*∇*η*; the wind forcing **F**; and the conservation of volume −∇ · (**u** *h*) are summarized as the right‐hand side function rhs. The time integration is now augmented with compensated summation. The rounding error *c*_*u*_ that occurs in the addition of the total tendency *du* to the previous time step *u*_*n*_ is calculated and stored. On the next time step, this rounding error is subtracted from the total tendency *du* in an attempt to compensate for the rounding error from the previous time step. This is illustrated here for the zonal velocity *u* in isolation, although in practice time integration has to update the prognostic variables *u*, *v*, *η* simultaneously. A compensated time integration for *u* with the fourth order Runge‐Kutta scheme can be written as

(4) $$\begin{array}{lll} k_{1} & = & \text{rhs}\left(u+\frac{\Delta t}{2}k_{1}\right)\\ k_{2} & = & \text{rhs}\left(u+\frac{\Delta t}{2}k_{2}\right)\\ k_{3} & = & \text{rhs}\left(u+\frac{\Delta t}{2}k_{3}\right)\\ k_{4} & = & \text{rhs}\left(u+\Delta tk_{3}\right)\\ du & = & \frac{\Delta t}{6}\left(k_{1}+2k_{2}+2k_{3}+k_{4}\right)\\ u_{n+1}^{*} & = & u_{n}+du\\ c_{u} & = & \left(u_{n+1}^{*}-u_{n}\right)-du \end{array}$$

with *c*_*u*_ = 0 as initial condition. The addition *u*_*n*_ + *du* usually suffers from rounding errors as described above. The loss of precision in *du* is calculated in *c*_*u*_ (which is only 0 in exact arithmetic). The compensation is analogously implemented with *c*_*v*_, *c*_*η*_ for the other prognostic variables.

The dissipative terms, that is, biharmonic diffusion of momentum *ν*_*B*_∇^4^ **u** and bottom friction −*r* **u**, are integrated with a single forward step after the Runge‐Kutta integration in ShallowWaters.jl and summarized as *rhs*_*diss*_. To compensate for rounding errors for both the dissipative and non‐dissipative terms simultaneously, *c*_*u*_ from Equation 4 is subtracted from the total dissipative tendency *du*_*diss*_. In that sense, the rounding error from Equation 4 is attempted to be compensated subsequently in Equation 5, and vice versa.

(5) $$\begin{array}{lll} du_{\text{diss}} & = & \Delta t{\text{rhs}}_{\text{diss}}\left(u_{n+1}^{*}\right)-c_{u}\\ u_{n+1} & = & u_{n+1}^{*}+du_{\text{diss}}\\ c_{u} & = & \left(u_{n+1}-u_{n+1}^{*}\right)-du_{\text{diss}} \end{array}$$

Only the addition of the total tendency is compensated here to minimize the amount of additional calculations, which increases when also compensating the four sub steps in the 4‐th order scheme.

The compensated time integration is an alternative to mixed‐precision approaches. While those aim to keep the precision high in the precision‐critical calculations, the compensated time integration introduces a new variable to compensate for the rounding errors in one precision‐critical calculation. With compensated time integration all variables can be kept in 16 bits, and no conversions between number formats are necessary.

## 5 A Fluid Simulation Calculated Entirely in 16 Bits

**Figure 3:**
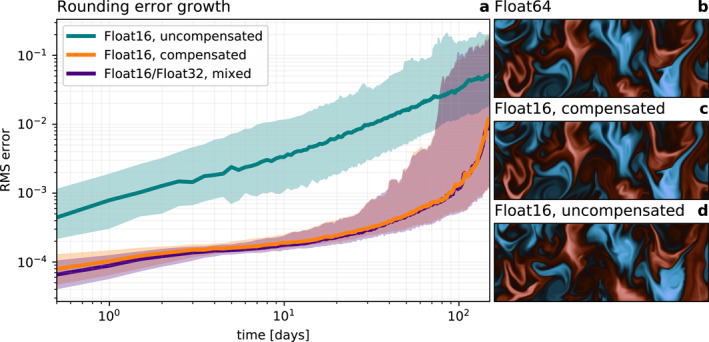
Rounding error growth with Float16 in ShallowWaters.jl using compensated time integration or mixed precision. (a) Errors are root‐mean square errors of zonal velocity *u* relative to Float64. Solid lines denote the median and shadings the interdecile confidence interval. (b and c) Snapshots of tracer *q* from a zoom into Figure 4 after 100 days of simulation and (d) as (c) but without compensated time integration.

The accumulated rounding error from mixing precision and compensated time integration is now assessed. ShallowWaters.jl is started from identical, in Float16 perfectly representable, initial conditions in a domain of 8,000 km by 4,000 km. The model is spun‐up to reach a turbulent flow domain‐wide, while the tracer starts from an idealized checkerboard pattern to better highlight the turbulence everywhere in the domain. The grid consists of 3,000 × 1,500 points at about 2.7 km grid‐spacing (see Appendix A for the physical parameters). With Float16 and without compensated time integration, the accumulated rounding error for zonal velocity *u* compared to Float64 exponentially increases 100‐fold in the first 150 days (Figure 3a). With mixed‐precision, using Float16 for the tendencies and Float32 for the prognostic variables, this rounding error growth is strongly reduced. After about 100 days (25,000 time steps) of integration, the errors with mixed‐precision are approximately equivalent to the errors without mixed‐precision after a few time steps. Beyond 100 days the error growth accelerates and chaos removes any information deriving from the initial conditions.

Using a compensated time integration, the rounding error from Float16 is strongly reduced and matches well with the error growth of mixed‐precision. From the perspective of rounding errors the two methods are therefore equivalently suited to reduce rounding errors with 16‐bit arithmetic. The rounding error growth of the other prognostic variables is similar. The positive effect of compensated time integration is well illustrated in snapshots of tracer mixing where even after 100 days of simulation only a very slight deviation from the Float64 reference is observable (Figures 3b–3d).

**Figure 4:**
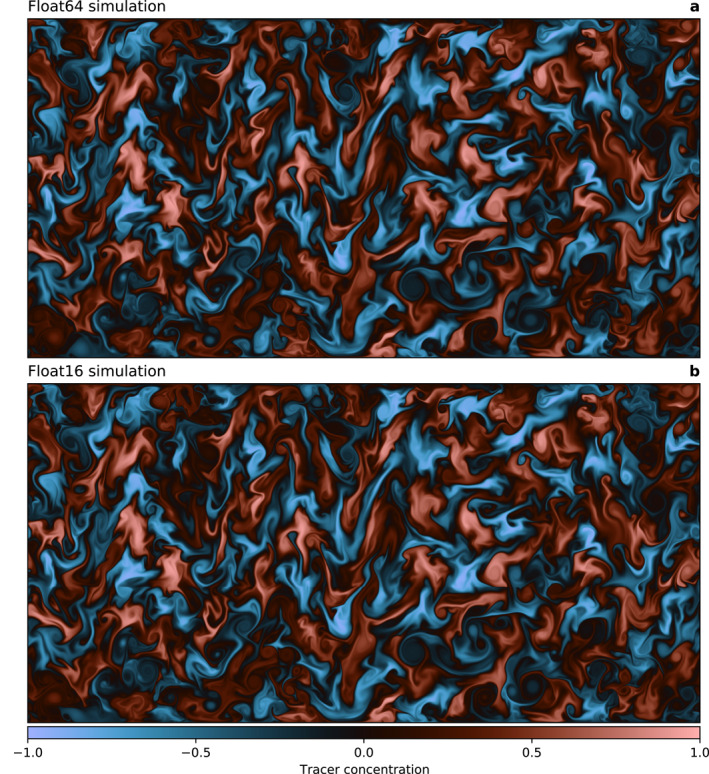
Turbulent tracer mixing as simulated by ShallowWaters.jl. (a) Simulation based on Float64 arithmetic and (b) Float16 with compensated time integration. Snapshot is taken after 100 days of simulation (about 25,000 time steps) with 3,000 × 1,500 grid points starting from identical initial conditions. Remaining errors between (a and b) from low‐precision Float16 are tolerable and will be masked by other sources of error in a less idealized model setup.

Even after 100 days a large simulation (3,000 × 1,500 grid points) with Float16 shows minimal errors in the tracer mixing compared to Float64 (Figure 4). Only at regions near the boundaries, where the mixing is enhanced, a difference is visible. The remaining rounding error is small and will be masked in a more realistic setup by model or discretization errors. To better understand the simulated timescales, an animated version of Figure 4 is available in the supplement.

Reducing the precision in calculations raises concerns about the numerical conservation of physically conserved quantities like mass. The compensated time integration conserves the mass in the shallow water equations with Float16 (<0.002% change within 500 days compared to Float64), similar to mixed precision (Figure S1). Without compensated time integration for Float16 the conservation is with 0.05% change over 500 days less accurate. Similar results were obtained for the conservation of the tracer. We will now assess the speedups with Float16 compared to Float64 on A64FX.

The A64FX is a microprocessor developed by Fujitsu based on the ARM‐architecture. It powers not just the fastest supercomputer in the world as of November 2021 (TOP500.org, Dongarra & Luszczek, 2011), Fugaku, but also a number of smaller systems around the world, including Isambard 2 which we use here. The A64FX has a number of features intended to accelerate machine learning applications. Notably, it allows not just Float32 and Float64 arithmetic but also Float16. Official benchmarks of the A64FX demonstrate a cost increase which is linear with the number of bits (FUJITSU, 2020; Odajima et al., 2020). In that sense, Float32 can be twice as fast in applications than Float64, while Float16 can be four times as fast, when optimized well. In practice, speedups in complex applications are due to a mix of factors: In compute‐bound applications, the wall‐clock time is largely given by the clock rate of the processor and the vectorization of arithmetic operations (such that small sets of them are performed in parallel on a single processor core). Using Float16 instead of Float64 allows to put four times as many numbers through the vectorization, theoretically allowing for 4x speedups. The performance of memory‐bound applications, on the other hand, is largely determined by the data transfer rate between the processor and its various levels of caches that increase in size but decrease in bandwidth. Using Float16 instead of Float64 allows to load four times as many numbers from memory, which theoretically translates to 4x speedup as well.

**Figure 5:**
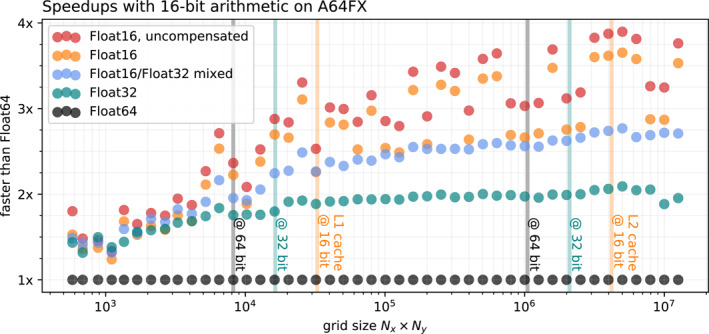
Performance increase from Float16 when running ShallowWaters.jl at varying grid sizes on A64FX. The grid size is the total number of grid points *N*_*x*_ × *N*_*y*_. All timings are single‐threaded median wall clock times relative to Float64, excluding compilation, model initialization and memory pre‐allocation. The corresponding size of the L1 and L2 cache (64KiB, 8 MiB) of A64FX is given as vertical lines for arrays of 16, 32, and 64‐bit floats.

ShallowWaters.jl is a memory‐bound application for which the biggest benefit from Float16 will be the reduction of the size of the arrays by a factor of four when compared to Float64. The arrays can therefore be read faster from memory with a potential speedup of 4x. We benchmark ShallowWaters.jl at varying grid sizes, excluding compilation, model initialization and memory pre‐allocation. With grid sizes of 10^5^ (about 450 × 225 grid points) and larger, there is a clear improvement from using Float32 instead of Float64 which approaches 2x speedups (Figure 5). Using Float16 these speedups reach up to 3.8x for grid sizes beyond 3 × 10^6^ (about 2,450 × 1,225 grid points). The dependency of the speedup on the grid size is complicated: While larger grids usually experience more acceleration on A64FX in Float16, there are ranges where the speedup drops to 3–3.25x. This is likely due to peculiarities in the memory and cache hierarchy of the A64FX, such that the performance benefit of Float16 cannot always be fully realized. A detailed assessment of these peculiarities is beyond the scope of this study, but it is nevertheless reassuring that, even in the worst case, Float16 is still at least 3 times faster than Float64 for these large grids.

As discussed in previous sections, using a compensated time integration can be used to minimize the rounding errors, which comes with a small additional computational cost: Using the compensated time integration the speedups drop to about 3.6x for large grids. Nevertheless, a compensated time integration yields higher performances than mixing the precision of Float16 and Float32, which approaches only 2.75x here. Consequently, a compensated time integration for Float16 is, although as precise, faster than mixed‐precision.

## 6 Conclusions

Low‐precision calculations for weather and climate simulations are a potential that is not yet fully exploited. While recently several weather forecast models moved toward Float32, 16‐bit arithmetic will likely find increasing support on future supercomputers. We present, to our knowledge, the first fluid simulation that runs entirely in hardware‐accelerated 16 bits with minimal rounding errors but at almost 4x the speed. The simulations were performed on A64FX, the microprocessor that powers Fugaku, the fastest supercomputer as of November 2021 according to TOP500.org.

The complex partial differential equations underlying weather and climate simulations are difficult to fit into the limited range of Float16. The shallow water equations considered here are not as complex as a three‐dimensional global weather or climate model. Whether the numbers in complex models can similarly be squeezed into the limited range of 16‐bit arithmetic remains unclear, but here we have introduced a method to do this more systematically. Sherlogs.jl is presented to analyze number formats and to evaluate the scaling of the equations. This technique allows to minimize under and overflows while making the most of the available representable numbers. In our case, subnormal floating‐point numbers had to be avoided and scaling of the equations dropped the amount of subnormals occurring below 0.2%.

Using 16‐bit floats will likely cause precision issues in fluid simulations. While mixed‐precision has been used to minimize rounding errors in precision‐critical calculations, we have presented here an approach that compensates for rounding errors to allow for simulations entirely within 16‐bit arithmetic. The compensated time integration minimizes rounding errors from this precision‐critical part of a simulation at a slightly higher cost. Benchmarking in comparison to mixed‐precision shows that the compensated time integration is faster in ShallowWaters.jl while being as precise as mixed‐precision.

The development of complex three‐dimensional models for 16 bits likely requires the techniques presented here to solve precision and range issues. It is possible that further scaling constants have to be introduced to better control the ranges in different parts of the model or in different horizontal layers. The compensated summation used here for the time integration may also be required in a different form in other model calculations like vertical integrations.

Alternatives to floats have been discussed for weather and climate simulations previously (Klöwer et al., 2019, 2020). Although posit numbers (Gustafson & Yonemoto, 2017) are more precise in these applications, the improvement from floats to posits is smaller than using mixed‐precision and therefore also smaller than the compensated time integration. In that sense, algorithms that are low‐precision resilient are far more important than the actual choice of the number format, especially given that only floats are widely hardware‐supported.

The work here shows that a naïve translation of the mathematical equations into code will likely fail with 16‐bit arithmetic. However, this does not mean that 16‐bit arithmetic is unsuited for the numerical solution of complex partial differential equations such as the shallow water equations. But it means that both precision and range issues have to be addressed in the design of the algorithms used. A compensated time integration is a low‐precision resilient algorithm, and scaling is essential to fit the very limited range of Float16.

While 16‐bit hardware is largely designed for machine learning, its potential to increase computational efficiency extends to weather and climate applications too. The techniques and results presented here show that 16‐bit calculations are indeed a competitive way to also accelerate Earth‐system simulations on available hardware. Whether all calculations can be performed with 16 bits remains an open problem. The development of 16‐bit models can be challenging, but would lead to a new generation of computationally efficient weather and climate models.

## Conflict of Interest

The authors declare no conflicts of interest relevant to this study.

## Supporting information

Figure S1

Movie S1

## Data Availability

Data and software to reproduce the analysis are available at Klöwer (2021a, 2021d). The software packages ShallowWaters.jl (v0.5) and Sherlogs.jl (v0.2) are available at Klöwer (2021b, 2021c).

## Appendix A

### A1. Model Setup

The domain *L*_*x*_ × *L*_*y*_ of the model setup of ShallowWaters.jl uses periodic boundary conditions in *x* and no‐slip at the northern and southern boundary. The standard depth is *H* = 500 m, and several meridional mountain ridges are placed at irregular distances on the seafloor to trigger instabilities in the flow. The layer thickness at rest is

(A1)
$$H\left(x\right)=H_{0}-\sum_{i}H_{1}\exp\left(-2H_{\sigma }^{-2}{\left(x-H_{x_{i}}L_{x}\right)}^{2}\right)$$

for *i* = 1, 2, 3, 4 with $H_{x_{i}}=\left(0.05,0.25,0.45,09\right)$ four relative *x*‐positions between 0 and *L*_*x*_. The height of the mountain ridges is *H*_1_ = 100 m and the characteristic width *H*_*σ*_ = 300 km. The fluid density is $\rho =1000\;\frac{kg}{m^{-3}}$, which inversely scales the wind forcing coefficient $F_{0}={\left(\rho H_{0}\right)}^{-1}F_{c}$ with *F*_*c*_ = 0.12 Pa. The biharmonic viscosity *ν*_*B*_ = Δ*x* ^2^ *ν*_*A*_ is derived from the harmonic viscosity *ν*_*A*_, which itself scales with the squared grid spacing Δ*x* ^2^, and uses *ν*_*A*,0_ = 500 m^2^s^−1^ at Δ*x*_0_ = 30 km.

(A2)
$$\nu_{B}=\Delta x^{2}\nu_{A}=\frac{\Delta x^{4}}{\Delta x_{0}^{2}}\nu_{A,0}$$

The linear bottom drag is disabled *r* = 0. The time step Δ*t* is chosen to resolve gravity waves, which propagate approximately at phase speed $c_{ph}=\sqrt{gH_{0}}\approx 2.2\;\frac{m}{s}$. With the Courant‐Friedrichs‐Lewy number (Courant et al., 1967) *C*_*FL*_ = 0.9, we use approximately $\Delta t=\frac{C_{FL}\Delta x}{c_{ph}}=17.9\;min$ for Δ*x* = 2.7 km. To reduce the diffusion in the tracer advection and for computational efficiency we increase the time step for the semi‐Lagrangian scheme to 12Δ*t*. For more details see Klöwer et al. (2020).

### A2. Floating‐Point Numbers and Subnormals

The 16‐bit floating‐point number format Float16 is with 1 sign bit, *n*_*e*_ = 5 exponent bits and *n*_*m*_ = 10 mantissa bits defined as

(A3)
$$x=\left\{\begin{array}{ccc} {\left(-1\right)}^{s}\cdot 2^{e-bias}\cdot \left(1+f\right)\;\; & \text{if}\;e>0, & \left(\text{normals}\right)\\ {\left(-1\right)}^{s}\cdot 2^{1-bias}\cdot f\;\; & \text{if}\;e=0\;\text{and}\;f>0, & \left(\text{subnormals}\right)\\ {\left(-1\right)}^{s}\cdot 0\;\; & \text{if}\;e=0\;\text{and}\;f=0, & \left(\pm 0\right)\\ {\left(-1\right)}^{s}\cdot \infty \;\; & \text{if}\;e=2^{n_{e}}-1\;\text{and}\;f=0, & \left(\pm \text{Inf}\right)\\ \text{NaN}\;\; & \text{if}\;e=2^{n_{e}}-1\;\text{and}\;f>0, & \left(\text{Not}‐A‐\text{Number}\right). \end{array}\right.$$

The five exponent bits are interpreted as an unsigned integer *e* ∈ {0, 1, …, 30, 31} and the subtraction with $bias=2^{n_{e}-1}-1=15$ converts them effectively into signed integers, representing positive or negative exponents. The mantissa bits form the fraction $f\in \left[0,1\right)$

(A4)
$$f=\sum_{j=1}^{n_{m}}m_{j}2^{-j}=\frac{m_{1}}{2}+\frac{m_{2}}{4}+\frac{m_{3}}{8}+\ldots$$

such that the state (*m*_1_ = 0 or 1) of the first mantissa bit determines whether $\frac{1}{2}$ is added to the sum, the second mantissa bit *m*_2_ determines whether $\frac{1}{4}$ is added, etc. The smallest subnormal number *minpos*, which is also the smallest representable number, occurs for all bits being 0 except the last mantissa bit *m*_10_ = 1, then *x* = 2^1−*bias*^ × 2^−10^ = 2^−24^ ≈ 6 × 10^−8^. The 1,023 positive subnormal numbers are linearly distributed between 0 and the smallest normal number, also called *floatmin*, *x* = 2^−14^ ≈ 6 × 10^−5^. The subnormals therefore fill the range between 0 and floatmin and were introduced to avoid underflows in some arithmetic operations with the smallest normal numbers. However, as they are an exception to the definition of normal floats, they also require special treatment on hardware, which reduces performance compared to the normal floats on some processors, for example, on ARM‐based A64FX.

The precision of Float16 throughout the range of representable numbers can be analyzed with the decimal precision *p* (Gustafson & Yonemoto, 2017; Klöwer et al., 2019, 2020).

(A5)
$$p\left(x_{\text{repr}},x_{\text{exact}}\right)=-\log_{10}|\log_{10}\left(\frac{x_{\text{repr}}}{x_{\text{exact}}}\right)|$$

*x*_exact_ is the exact result of an arithmetic operation and *x*_repr_ the closest representable number in a given format and $\mathrm{sign}(x_{\mathrm{exact}})=\mathrm{sign}(x_{\mathrm{repr}})\neq 0$. An arithmetic result that lies halfway between two representable numbers experiences the worst‐case rounding error, which determines the worst‐case decimal precision for that number. An overview of decimal precisions for Float16 and Float32 is given in Figure 1.
