# Supplementary material for: Fluid Simulations Accelerated With 16 Bits: Approaching 4x Speedup on A64FX by Squeezing ShallowWaters.jl Into Float16
Source: J Adv Model Earth Syst. 2022 Feb 11;14(2):e2021MS002684. doi: 10.1029/2021MS002684 (PMC9287017; doi:10.1029/2021MS002684)
Supplement: Supplementary file 1 — Figure S1 [file JAME-14-0-s001.pdf]

AGU PUBLICATIONS

Journal of Advances in Modeling Earth Systems

Supporting Information for

Fluid simulations accelerated with 16 bits: Approaching 4x speedup on A64FX by squeezing ShallowWaters.jl into Float16

Milan Klöwer[1], Sam Hatfield[2], Matteo Croci[3], Peter D. Düben[2] and Tim N. Palmer[1]

[1]Atmospheric, Oceanic and Planetary Physics, University of Oxford, Oxford, UK
[2]European Centre for Medium-Range Weather Forecasts, Reading, UK
[3]Mathematical Institute, University of Oxford, Oxford, UK

Contents of this file

Figure S1

Introduction

The supporting information provides an additional figure that illustrates the conservation of mass and tracer in the simulations described in the main article.

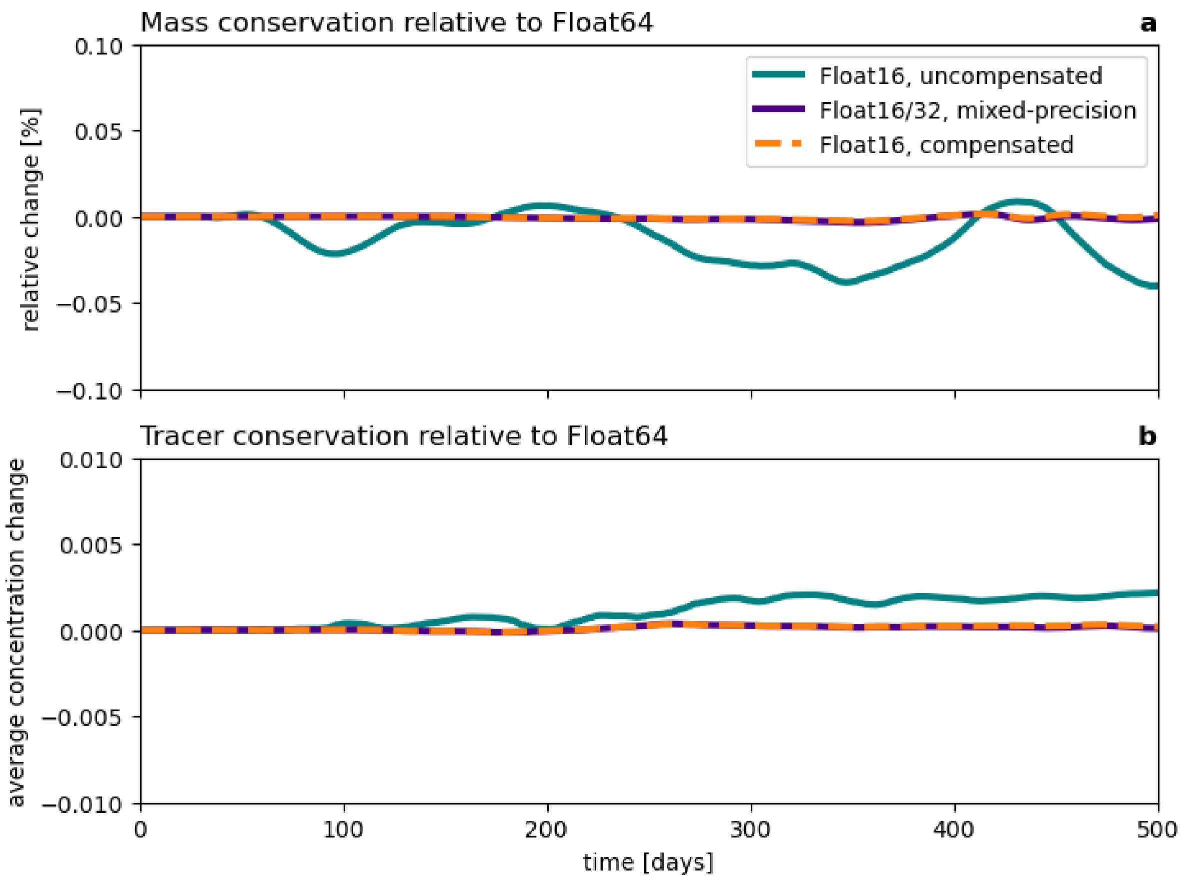

Figure S1. Mass and tracer conservation with Float16 arithmetic**. a** Mass conservation relative to Float64. **b** Tracer conservation relative to Float64 in units of tracer concentration with initial conditions in (-1,1), see Fig. 5. Both mass and tracer are well conserved with Float16 arithmetic. Best conservations are obtained with compensated time integration or mixed-precision.
